# The CONTEXT trial: Real world clinical use of GIQuant—a motility MRI based activity biomarker for small bowel Crohn’s disease

**DOI:** 10.1093/ecco-jcc/jjag037

**Published:** 2026-06-10

**Authors:** Alex Menys, Iyad Naim, Joy Barber, Kamal Patel, Nirav Patel, Attah Ocholi, Samantha Baillie, Nkem Onyeador, Victoria Jennings, Sudheer Vuyyuru, Christopher Clarke, Rahul Munyal, Gauraang Bhatnagar, Thomas Shepherd, Laurence Bourn, Sara Upponi, Juan De la Revilla Negro, Anish Kuzhiyanjal, Jimmi Limdi, Aung Win, Priyanka Ghodekar, Afaq Siddiqui, Damian Tolan, Heather Fitzke, William Weston, Paul Harrow, Jie Han Yeo, M Hameed, Stuart A Taylor, Gordon William Moran

**Affiliations:** Motilent, London, United Kingdom; Centre for Medical Imaging, University College London, London, United Kingdom; Motilent, London, United Kingdom; St George’s Hospital, Gastroenterology, London, United Kingdom; St George’s Hospital, Gastroenterology, London, United Kingdom; St George’s Hospital, Gastroenterology, London, United Kingdom; St George’s Hospital, Gastroenterology, London, United Kingdom; St George’s Hospital, Gastroenterology, London, United Kingdom; St George’s Hospital, Gastroenterology, London, United Kingdom; Nottingham University Hospitals, Nottingham, United Kingdom; Nottingham University Hospitals, Nottingham, United Kingdom; Nottingham University Hospitals, Nottingham, United Kingdom; Nottingham University Hospitals, Nottingham, United Kingdom; Frimley Park Hospital, Frimley, United Kingdom; Frimley Park Hospital, Frimley, United Kingdom; Motilent, London, United Kingdom; Addenbrooke’s- University of Cambridge, Gastroenterology, Cambridge, United Kingdom; Addenbrooke’s- University of Cambridge, Gastroenterology, Cambridge, United Kingdom; Northern Care Alliance NHS Foundation Trust, Manchester, United Kingdom; Northern Care Alliance NHS Foundation Trust, Manchester, United Kingdom; Northern Care Alliance NHS Foundation Trust, Manchester, United Kingdom; Northern Care Alliance NHS Foundation Trust, Manchester, United Kingdom; Northern Care Alliance NHS Foundation Trust, Manchester, United Kingdom; Leeds Teaching Hospitals NHS Trust, Leeds, United Kingdom; Centre for Medical Imaging, University College London, London, United Kingdom; Centre for Medical Imaging, University College London, London, United Kingdom; Department of Gastroenterology, University College Hospitals London, London, United Kingdom; Department of Gastroenterology, University College Hospitals London, London, United Kingdom; Centre for Medical Imaging, University College London, London, United Kingdom; Centre for Medical Imaging, University College London, London, United Kingdom; Nottingham University Hospitals, Nottingham, United Kingdom

## Abstract

**Background:**

Motility MRI is a validated marker of small bowel Crohn’s disease (CD) activity. Objective assessment of small bowel motility with GIQuant (Motilent, London, U.K.) may have a valuable role to play in personalised patient care. The aim of this study was to assess the impact of implementing GIQuant on clinical impressions around treatment response in small bowel CD.

**Methods:**

Patients with small bowel CD on or starting therapy underwent two MRE scans (∼1 year apart) including motility MRI (mMRI) imaging. Twelve gastroenterologists from six U.K. participating centres evaluated patient response based on standard of care tests, grading them on a 6-point scale (*much/somewhat/slightly* worse through to *slightly/somewhat/much* better). After initial assessment, GIQuant scores and percentage change were revealed to the gastroenterologist who then updated their response assessment with this information. GIQuant scores at baseline and follow up are reported along with magnitude of change in clinically categorised responders and non-responders.

**Results:**

Over the study period of 3.6 years a total of 253 patients (61% male), with ileal (52%) or ileocolonic (48%) CD underwent 506 MRE scans, with a median 10.4-month gap (range 2 to 36) between scans.

In 120/253 (47%) patients, gastroenterologists reported a change in their impression of patient status (one step change or greater) after reviewing the GIQuant results with 55/253 (22%) reversing their overall assessment (between deterioration or improvement) across the two time points.

In clinical responders, the GIQuant score increased from 123 to 163 (32% increase), *P* < .001 with non-responders showing a 2 point decrease in score (*P* = .9)

**Conclusion:**

GIQuant as a measure of motility MRI demonstrated robust utility to track the response of small bowel Crohn’s activity to therapeutic intervention across time points.

## Graphical abstract

**Graphical Abstract:**
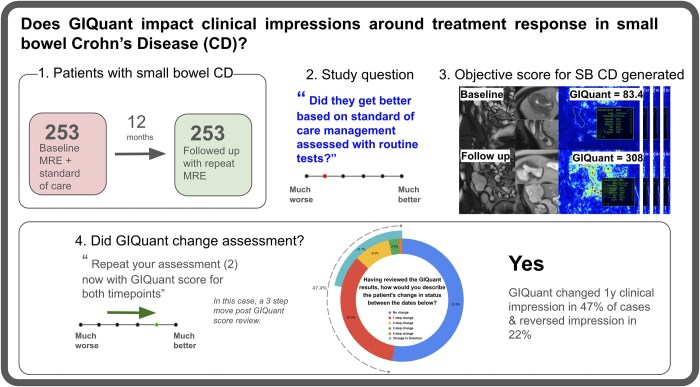

## Introduction

Crohn’s disease is a chronic, relapsing, and remitting immune-mediated inflammatory disease affecting the GI tract^1^. Modern and evolving definitions of treatment targets acknowledge the need to treat beyond symptoms to the target of mucosal and transmural healing as outlined by the Selecting Therapeutic Targets in Inflammatory Bowel Disease (STRIDE)^2^ and the most recent European Crohn’s and Colitis Organisation guidelines (ECCO)^3^ guidelines. Selecting the optimal approach for transmural disease assessment remains challenging, being influenced by a number of variables including disease location, behaviour, extraintestinal manifestations and patient preference. Nearly 50% of CD patients have small bowel involvement^4^, and given its relative inaccessibility, non-invasive cross-sectional imaging modalities like MRI and intestinal ultrasound are essential to management. Currently disease activity assessment hinges on the evaluation of structural features e.g. wall thickness. Despite numerous indices being available, none are used routinely in clinical practice as part of a precision medicine approach^5^.

Even where cross sectional imaging is routinely used, structural changes may lag behind clinical and endoscopic improvements. Furthermore, distinguishing between fibrotic and inflammatory segments remains a critical, unanswered “decision point” in treatment and both processes may be indistinguishable on conventional MRE and ultrasound. To provide a greater information yield, modern MRI techniques combined with post-processing software can assess functional parameters in an objective manner, specifically peristalsis^6^. A reduction in motility accurately reflects inflammatory disease activity^7^ and may normalise in response to effective medical treatment^8^. mMRI and the corresponding image analysis technology under assessment here, GIQuant (Motilent, London, U.K.), enables objective score extraction from routine data. GIQuant has made significant advances along the imaging biomarker development pathway, including rigorous technical validation and extensive clinical testing^10^^,^^11^^,^^12^^,^^13^^,^^14^, in multicentre prospective trials^6^^,^^7^^,^^8^. However, imaging biomarkers, like any other biomarker, require assessment of clinical impact, behavioural change and cost-effectiveness^9^ after technical, biological and initial clinical validation.

An important next step in the adoption of any new biomarker is successful integration into existing clinical pathways, and a demonstrable potential positive impact on patient care prior to complex and expensive interventional clinical trials. One way to assess this is to integrate the new biomarker in the clinic, evaluate ease of use and assess impact on clinical evaluation in routine care. Indeed, many promising biomarkers fail at this important step especially against an evolving diagnostic landscape which is particularly pertinent in IBD with the rise of intestinal ultrasound delivered in the point of care setting^15^^,^^16^^,^^17^. To date there has been no such “real world” evaluation of mMRI or GIQuant and how clinicians may integrate the information it gives on their diagnostic confidence and decision-making process.

We know there is a clinical challenge around the assessment of small bowel CD especially in patients undergoing treatment and to evaluate the potential utility of GIQuant, in this study we integrated objective scoring into existing CD care pathways and modelled the impact on clinical decision at six high-volume U.K. hospitals, otherwise naive to this technology.

## Materials & methods

### Study synopsis

The aim of this study was to acquire real world clinical impressions around treatment response before and after seeing the GIQuant (Motilent, London, U.K.) score. GIQuant provides a quantitative measure of small bowel motility as a marker of bowel health. As such, this study was limited to *small bowel* Crohn’s where peristalsis is consistent under the influence of luminal contrast in routine MRE exams. Our aims were to assess:

1. If GIQuant values in the real world setting are consistent with previously reported literature values in diseased bowel.
2. Whether a quantitative GIQuant score for small bowel CD activity and treatment response within 1 year influences the clinical impression of the treating gastroenterologist (i.e. has the patient’s small bowel disease improved or worsened?).
3. If there are specific disease phenotypes in which GIQuant had particular utility.
4. If, upon viewing GIQuant scores, there was any proactive impact on gastroenterologists' treatment decision making.

Users were familiarised with the technology prior to assessing its clinical impact

### Technology under investigation

**Figure 1.**
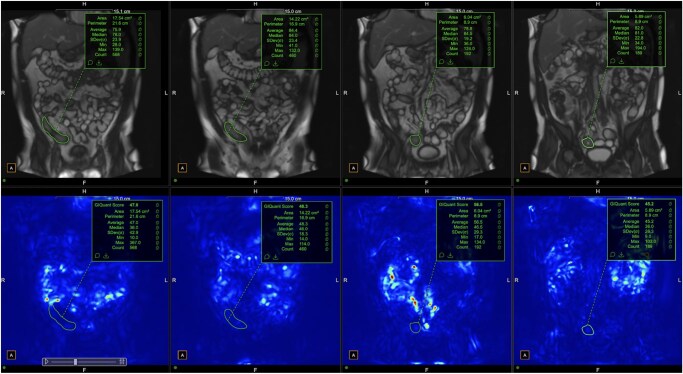
A single subject with a 29 cm terminal ileal lesion. The disease covers multiple slice locations where cine imaging was collected. A GIQuant reference image (top) and motility map (bottom) is created for every anatomical position, four of which are shown above. While the GIQuant score is relatively consistent, the bottom left image was used to produce the lesion score due to it having the largest area.

GIQuant quantifies bowel wall motion referred to here as motility (and not transit), generating a numeric measure of cine or dynamic mMRI datasets. The technology is available as a Class IIa medical device in the U.K./EU and FDA 510k cleared in the United States. Active small bowel CD is associated with reduced peristalsis, with a return of peristalsis being considered as a marker of treatment response. The GIQuant score is “unitless”, with the standard deviation of pixel movement in the underlying time series with motion estimated using a validated registration algorithm. A GIQuant score of < 230 and < 220 is proposed to reflect endoscopic and histological disease activity respectively^7^, with scores < 150 often seen in highly active disease and over 300 generally considered normal in the context of small bowel CD^6^^,^^18^. To generate a GIQuant score from mMRI cine motility images, a radiologist or suitably trained professional, manually places a Region of Interest (ROI) on a reference image in the segment(s) of small bowel of interest. This ROI is then manually copied or automatically propagated to a parametric motility map where the average pixel intensity under the ROI is the GIQuant score (Figure 1). GIQuant values are multiplied by 1000 for consistent and robust display on hospital PACS systems but the scores may be divided by 1000 to relate to historical metrics generated under research implementation of the algorithm^7^.

GIQuant may be quantitatively assessed at a single time point but is more commonly used longitudinally to evaluate changes in bowel peristalsis alongside other morphological measures e.g. bowel wall thickness. GIQuant scores are either screenshot as a key image within the hospital PACS and/or the GIQuant score described in part of the radiologists dictated report text, akin to bowel wall thickness, where it can then appear in the electronic patient records.

### Study design and methods of data collection and data analysis

This was an observational multi-centre prospective technology perception study with no randomisation. The study was conducted in accordance with national ethical permissions (21/PR/0592) and each site was assessed for capacity and capability.

## Study site eligibility criteria

### Primary sites

- Ability to technically install the GIQuant software within the clinical care setting.
- Motility MRI or 'cine' imaging not part of routine MRE protocols but being introduced during the study period
- Intravenous gadolinium injection part of routine MRE protocols

### Secondary sites

- Motility MRI or 'cine' imaging is already part of routine MRE protocols.
- Intravenous gadolinium injection part of routine MRE protocols.

### Exclusion criteria (primary and secondary sites)

- Inability to access REDCap eCRF on hospital computers or via WiFi.
- Inability to support GIQuant technically (e.g. no Virtual Machine provision, no IT support, PACS ROI tools unable to extract GIQuant score)

### Clinician inclusion criteria

- Ability to give informed consent.
- Consultant Gastroenterologist or Gastrointestinal Radiologist (on GMC specialist register) in the National Health Service, UK.
- Agreement to comply with the study training requirements, protocol and regulatory/sponsor stipulations (including GIQuant & eCRF training).

### Target patient population

All patients had known or suspected small bowel CD at the time of their first MRE scan and were undergoing disease assessment with a second follow up MRE scan, typically within 12 months on institution scanner(s) (Appendix 3). Both MRE scans were required to have cine motility imaging. The follow up MRE was scheduled as part of their routine care and participating gastroenterologists were asked to manage all patients as per their institution’s usual standard of care. The majority of patients were expected to initiate or switch at least one advanced therapy over the study period. Primary sites introducing cine MRI to their MRE protocol could identify potential patients prospectively and await their follow up scan. Secondary sites, who had historical motility MRE data which could be processed with GIQuant, could identify patients at the time of the follow up MRE and use their most recent historical scan as the baseline (again typically within the 12 months prior).

## eCRF completion

### Radiologist derivation of the GIQuant score

**Figure 2.**
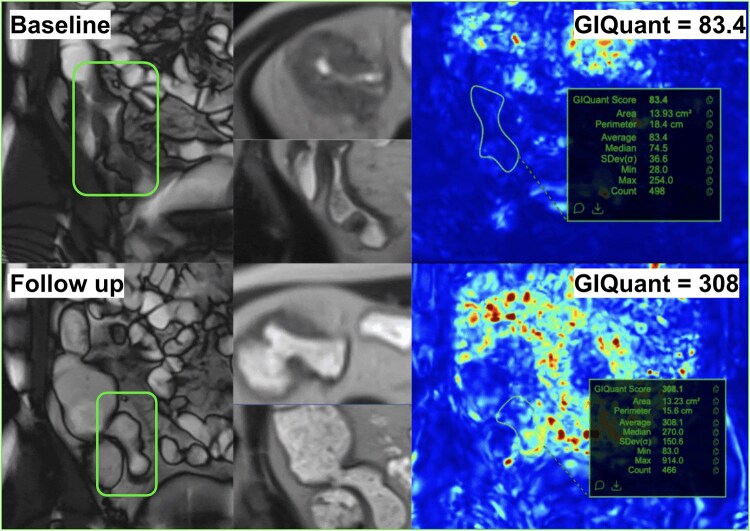
A rigid non-peristalsing TI stricture improves and regains full functionality. Visually there is an improvement in active inflammation with GIQuant improving from 83.4 to 308.1.

To generate a GIQuant score, a radiologist with a subspeciality interest in gastrointestinal imaging reviewed the patient’s MRE, identified the disease location and classified the phenotype as inflammatory, stricturing or penetrating. They then selected a small bowel segment on the baseline MRE (the segment they considered most active based on conventional morphological observations) e.g. the terminal ileum and located the same loop on the follow up MRE dataset. Using the GIQuant Reference images (Figure 2) they placed a polygonal ROI within the selected small bowel loop. Radiologists were made aware of the impact of oral contrast on motility (distended bowel is more peristaltically active than fasted)^19^ and we advised all readers to look for contrast proximal and distal to the lesion under investigation. The slice with the greatest disease area within a selected loop visible across multiple slices in the GIQuant Reference Image volume, was selected. The two MRE scans were examined side by side to best match the selected segment at both time points to ensure comparable observations, akin to RECIST reporting of oncological imaging. This method for lesion selection is described in further detail in Plumb et al. 2024^12^. With the Reference image selected, the ROI was transferred either through copy and paste or propagation (depending on PACS behaviour) to the GIQuant Map. The average pixel intensity under the GIQuant Map is the GIQuant score for that lesion and was recorded on an electronic CRF in REDCap. All radiologists were encouraged to familiarise themselves with GIQuant in routine clinical practice, though cases identified for the study by the gastroenterologist which were omitted from evaluation with GIQuant during this familiarisation period to avoid bias. An extensive prior literature has demonstrated good/very good agreement between radiologists for GIQuant measurement in adult^6^^,^^10^^,^^12^^,^^13^^,^^14^ and paediatric^13^^,^^14^^,^^20^ populations even in those inexperienced in using the software^10^. As such, additional evaluation of interobserver variability was not performed as part of this study. In routine clinical practice, the GIQuant score would be dictated and captured in the radiologist report and/or screen shot and saved as a key image.

### Gastroenterologist training

Prior to commencing the study, each participant was required to complete an online consent form and participant information eCRF via REDCap. This form collected details including age range, professional role, years of experience, prior experience with GIQuant, and any conflicts of interest (Appendix II).

A clinical assessment eCRF was designed by the study investigators (Appendix II) to systematically capture gastroenterologists' clinical impression and diagnostic confidence around treatment response and investigate how this assessment was impacted by the addition of objective information in the form of the GIQuant score including whether the results would have theoretically altered their management plan. One of the key questions explored the gastroenterologist assessment in *“how would you describe the patient’s change in status between the dates below?*” Response options (see below) were formulated to ensure standardization, clarity, and clinical relevance, minimizing subjectivity in data collection. Secondary questions were asked and presented as supplementary data (Appendix II).

Online or in-person training was provided for all participating gastroenterologists along with a summary of the literature and study aims. Primary sites, adopting both mMRI and GIQuant were given six months to familiarise themselves with the updates to their MRI service and further discuss GIQuant with their radiology colleagues. All participants completed an approximately 30-minute GIQuant and REDCap training to familiarise themselves with the structured reporting template and were required to complete at least 5 test eCRFs before starting the study to become familiar with the study workflow.

### Gastroenterologists interpreting patients with GIQuant

Part 1 of the eCRF: After identifying a patient within the target population (known or suspected small bowel CD with two MRE scans), additional demographic details were recorded including disease duration, smoking history, prior bowel surgeries, and current treatments (e.g. corticosteroids, immunomodulators, advanced therapies). Disease was classified in line with the Montreal classification^21^ for adults and Paris classification^22^ for paediatric patients, and use of standard of care tests including endoscopy, other imaging, blood and stool tests in the interval between the 2 MRES were recorded.

Once identified, the site study coordinator would pass on the patient details to the Radiologist to read each of the paired MREs, including recording of the GIQuant score, with the results transcribed into Part 3 of the eCRF.

Part 2 of the eCRF: Each gastroenterologist reviewed all the available clinical data collated as part of usual clinical care between the time of the 2 MREs including clinic letters, patient symptoms, endoscopy findings, C-reactive protein (CRP), faecal calprotectin, treatment history, and imaging (including the standard clinical reports of the two MRE scans–excluding GIQuant scores) before answering the study questions on a six-point scale (see CRF appendix II). The questions focused on the change in patients status between MRE scans, for example:

“Based on available tests, describe the change in patient’s status between the two MREs:

*Much worse/ Somewhat worse/ Slightly worse/ Slightly better/ Somewhat better/ Much better.”*

Part 3 of the eCRF: The study coordinator would now grant the gastroenterologist access to GIQuant scores for the patient. Here the same question(s) as Part 2 were asked with the prefix “Having reviewed the GIQuant results, …” The eCRF was then completed and submitted for review.

Two additional questions on Gastroenterologists evaluation were additionally asked pre and post GIQuant scores regarding i) 12 months prognosis which was scored on the same six-point scale and ii) likelihood of surgery within the next 12 months which was scored on six- point likelihood scale ranging from Definitely to *not* Definitely.

A final question was asked about impact on treatment plan to capture actual decisional changes based on the GIQuant score. As a novel tool we did not anticipate any changes.

### Statistical analysis

The goal of this study was to acquire real world clinical impressions around treatment response before and after seeing the GIQuant score. Our aim was to assess the change in gastroenterologist impression with the addition of new data. Importantly there was not a 'right or wrong' direction per se but a change would imply GIQuant was having some effect on gastroenterologists' assessment. Using a 6-point Likert-like scale (much worse through to much better) we expected to see a small -modest change in impression (and assumed exposure to a novel score would not radically change clinical assessment). Based on pilot data we expected a 1.5 decision unit standard deviation. At 80% power, with significance at 0.05 we needed approximately 200 pairs of measurements to see a small effect (0.3) using the two-tailed Cohen’s D test (0.2 = small, 0.5 = moderate and 0.8 = large difference between groups). 200 pairs of datasets was also practical with a target of six sites and 10 gastroenterologists. Cohen’s D (mean difference divided by pooled standard deviation) was calculated for the study population and reported. Data was presented visually to inspect direction and magnitude of change. Group level statistics e.g. GIQuant scans at time baseline and follow up were compared using paired T-test with median, ranges and standard deviations presented. All data was extracted from eCRFs and tabulated demographics, medication and disease classification. Statistical analyses were conducted using Python (scikit-learn, NumPy, Matplotlib, and Pandas).

## Results

### Patient demographics

Six UK sites were initiated: two primary with no experience with mMRI or GIQuant and three secondary who had experience of using mMRI, one of which had a GIQuant installation before the study commenced. Twelve gastroenterologists (5 from Primary sites) and twelve radiologists (8 from Secondary sites) participated. Radiologists responsible for computing the GIQuant^®^ score had a median of 5.5 years of experience of MRE, while gastroenterologists had a median of 4 years of experience managing CD.

Patient demographics are summarised in Table 1. Over the study period (3.6 years) a total of 253 patients (61% male) with ileal (54%) or ileocolonic (46%) Crohn’s disease underwent 506 MRE scans, with a median 10.4 month gap (range 2 to 36) between scans. At the time of the first MRE, 134 had disease duration > 4 years, 34 patients 3–4 years, 40 with 1–2 years, and 45 patients < 12 months. The majority of patients (207) were on a therapy at the time of their first scan most commonly with infliximab (70), adalimumab (63) and ustekinumab (46) (Table 2). 66 of 253, (26%) had CD-related bowel surgery at the time of their first MRE and an additional 38 had already been referred for surgery at the time of their first scan.

**Table 1.** *Patient classification*.

Classification	No. of patients
**Montreal classification, adult patients**	237
**Age at diagnosis:**	
**<17 (A1)**	40
17–40 (A2)	130
**>40 (A3)**	67
**Location of disease:**	
**Ileal (L1)**	130
**Ileo-colonic (L3)**	107
Behaviour of disease:*	
**Non-stricturing, non-penetrating (B1)**	99
**Stricturing (B2)**	91
***Penetrating (B3)***	46
**Paris classification, 16 paediatric patients (age < 16)**	16
**Age at diagnosis:**	
**10 - <17 (A1b)**	10
**0 - <10 (A1a)**	6
**Location of the disease**	
**Ileocolonic (L3)**	10
**Distal 1/3 ileum ± limited caecal (L1)**	6
Behaviour of disease:*	
**Non-stricturing, non-penetrating (B1)**	13
**Stricturing (B2)**	3

**Table 2.** *Medication status*.

Therapy	No. of patients	Duration (months)
**Adalimumab**	63	18.43 ± 20.59
**Infliximab**	70	19.99 ± 21.49
**Upadacitinib**	13	4.14 ± 1.68
**Ustekinumab**	46	17.43 ± 14.53
**Vedolizumab**	15	22.67 ± 19.00

Gastroenterologists had access to colonoscopy (79/253), blood tests (including: CRP, blood count, Urea & Electrolytes and creatinine), (243/253), faecal calprotectin results (143/253) or symptoms scores (96/253) at the time of the first MRE and colonoscopy (42/253), blood tests (242/253), faecal calprotectin results (124/253) and symptoms scores (97/253) around the time of the second MRE (Table 3).

**Table 3.** *Tests performed within three months of standard of care assessment*.

Tests performed within 3 months of each MRE	First MRE	Second MRE
**Colonoscopy**	79	42
**CT enterography**	2	1
**Capsule endoscopy**	0	2
**Harvey Bradshaw Index**	96	97
**Blood tests (including CRP)**	243	242
**Faecal Calprotectin**	143	124
**Abdominal Ultrasound**	15	9
**Enteroscopy**	1	0
**Other**	29	27

### GIQuant values across the cohort

The median GIQuant score at baseline was 123 (Sdev = 82, range 27 to 500) and at follow-up was 143 (Sdev = 107, range, 41 to 798) with a cohort increase of GIQuant 20 units (*P* < .001) between scans showing an upward trend in segment motility over time.

After initial review of clinical data *without* GIQuant, the cohort was split into responders (n = 147: *Slightly better/Somewhat better/Much better*) indicating an improvement in clinical assessment, regardless of magnitude) and non-responders (n = 106: *Much worse/Somewhat worse/Slightly worse)* indicating a deterioration in clinical assessment, again, regardless of magnitude).

Among responders, there was a significant increase in GIQuant score of 40 GIQuant units (*P* < .001) between the baseline (median = 123, Sdev = 78, range 27 to 480) and follow up MRE median 163, Sdev = 108, range 44 to 798).

In non-responders there was a non-significant decrease of -2 GIQuant unit (*P* = .9) between first (median = 125, Sdev = 89 range 41 to 500) and second (median = 123, Sdev = 102, range 41 to 774) MRE.

### Impact of GIQuant on gastroenterologist impression

**Figure 3.**
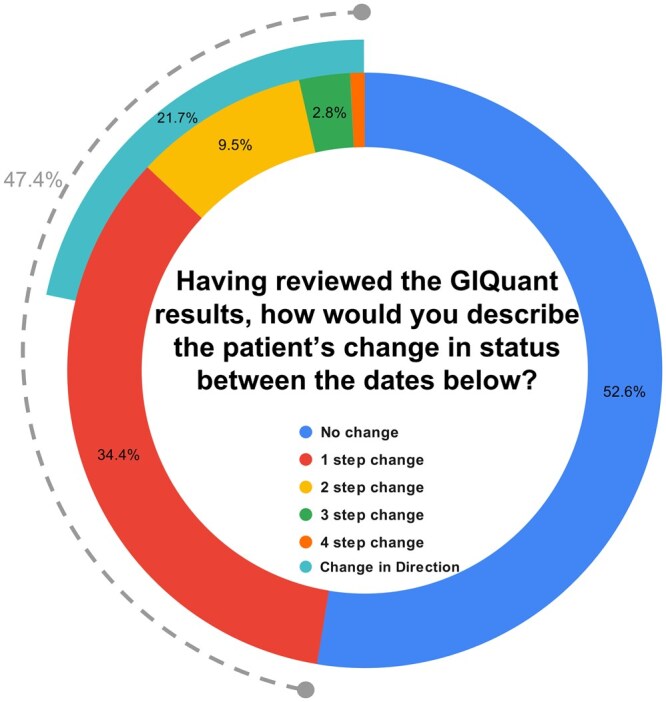
Change in gastroenterologists' clinical impression before and after reviewing the GIQuant scores.

Overall, Gastroenterologists reported a change in their impression of patient status (one step change or greater) after reviewing the GIQuant^®^ results in 47.4% (120/253) of cases with 21.7% (55/253) showing a reversal (e.g. from Somewhat worse to Slightly better) in their overall assessment (Figure 3). Across the cohort, the mean absolute change in score was 0.65 with a standard deviation of 0.82 producing a Cohen’s D = 0.79 which translates to a moderate to strong effect size.

The magnitude of impression change appeared not to be related to a particular sub-stype of small bowel Crohn’s Disease (Table 4).

**Table 4.** *Breakdown of gastroenterologist impression by disease phenotype*.

	Change in Gastroenterologists impression as a 1 or more step move (%)	Reversal in Gastroenterologists impression	GIQuant score at time point 1 (baseline)	GIQuant score at time point 2 (follow up)	Median change in GIQuant score between baseline & follow up
**Non-stricturing, non-penetrating (B1)** **n** = **112**	55 (49.1%)	23 (20.5%)	127 (45–480)	157 (55–718)	26 (–197 to 330)
**Stricturing (B2) n** = **94**	42 (44.7%)	21 (22.3%)	113 (27–500)	128 (41–798)	18 (–246 to 410)
**Penetrating (B3) n** = **46**	23 (50.0%)	11 (23.9%)	123 (46–248)	117 (44–450)	5 (–135 to 347)

With respect to Gastroenterologists assessment of patient prognosis, 36% (91/253) of cases had a change of impression with 16.2% reversal in progression direction (ie, better to worse or vice versa). For likelihood of surgery in the next 12 months, 24.7% (41/253) of cases showed a change in impression, and 10.7% (23/215) showed a change in direction of that impression—9 (4.2%) shifted toward greater surgical likelihood and 14 (6.5%) toward lower likelihood (full results in Appendix I).

There were no reported changes in actual treatment plans before and after reviewing the GIQuant scores.

## Discussion

The CONTEXT study is the first real world study of both small bowel motility and the GIQuant software to quantify small bowel CD activity focusing specifically on Gastroenterologists assessment in a cohort of 253 patients. In this study we found, first, that GIQuant ranges in this real world setting were consistent with previously reported results/values^6^^,^^7^^,^^8^^,^^11^^,^^12^^,^^13^^,^^14^. Second, clinical responders showed a significant improvement in GIQuant score in line with previously reported studies and our understanding of treatment response in the small bowel. Third, when presented with the GIQuant score, gastroenterologists frequently changed their assessment of the patient’s clinical response and prognosis indicating the need for and an interest in GIQuant as a small bowel disease activity marker.

Establishing clinical ranges represents the cornerstone of biomarker development together with a contextual understanding of how this new marker fits in with established tests^9^. Historically, endoscopic and histological activity with GIQuant had a threshold of approximately 230 units^7^ with fibrostenotic lesions scoring 150 in previous studies^23^^,^^24^. Recent Society of Abdominal Radiology (SAR) guidelines^25^ drew an aggregate threshold of 200 as being abnormal with normal reference ranges over 300^6^^,^^18^. In the CONTEXT study, our patient cohort was skewed towards those on, changing or starting therapy that required follow up. Across the cohort, the median GIQuant score was low at 123, as expected, showing broad agreement across a range of centres in the U.K. Statistically, there was a positive increase in GIQuant score between scans but naturally, not all the patients were improving in that time. Dichotomising the cohort in those improving (based on available clinical tests) from those not, showed a larger increase in GIQuant across the two time points of 40 units (from 123 to 163). Additional correlates against metrics including endoscopy, histology or inflammatory markers were not repeated here given the prior literature and added burden that this would have placed on clinical workup that nevertheless represents a limitation here. A future step would be to explore how a marked improvement in GIQuant score (and recovery of small bowel motility) impacted long-term outcomes and ultimately, if a treat-to-target approach using GIQuant could help rationalise therapeutic strategy e.g. rapid advancement to surgery in burnt out, fibrotic disease with no evidence of motility. These results should also be interpreted with the support of usability data across a range of populations and user experience levels displaying good inter-reader agreement as well as application in adolescent and pediatric cohorts^12^^,^^13^^,^^14^. In summary, across a range of sites and radiologists, GIQuant scores appeared consistent (in terms of ranges, directional change and absolute numbers) with previously published studies and an increase in the GIQuant lesion score agreed with gastroenterologists assessment of patient status.

CONTEXT, is the first study to explore the impact of GIQuant on gastroenterologists' impression at the one year follow up. Adopting a pragmatic approach, we asked practicing gastroenterologists to look first at their routine clinical tests available to them at their institution to make a subjective assessment of response. We then added the GIQuant score and percentage change and asked them to reassess, with our primary question focusing on response at one year. We found, surprisingly, that 47% of cases saw a change in a six-point impression scale and in 22% this constituted a reversal in direction e.g. somewhat better to somewhat worse implying that the addition of the GIQuant score was impacting their clinical assessment. To our knowledge, this is the first time that such an approach has been used to evaluate an imaging biomarker.

Our study has limitations. Although we asked treating gastroenterologists to provide a clinical impression from the GIQuant scores, they were not mandated by study protocol to alter treatment decisions solely on the basis of GIQuant scores. Consistent with this, there were no reported changes to treatment plans after reviewing the GIQuant scores, although future studies should explore protocol driven treatment adjustments. For example, establishing a baseline score and expected change to distinguish fibrosis from inflamed segments under the hypothesis that motility (and therefore the GIQuant score) will never increase where a bowel is heavily impacted by fibrosis. Second, we did select for a phenotype where, arguably, there is the greatest uncertainty in clinical evaluation. Anatomical challenges posed by small bowel CD with often an extensive transmural component makes endoscopy and histology challenging outside of assessment of surgically resected segments). This may arguably have added a degree of subjectivity to gastroenterologists' assessments of values provided by GIQuant scores. Yet, our limitations underpin the need for objective tests for small bowel disease along with efficacious strategies especially to address clinical ambiguities with assessment of fibrostenotic CD. We did not conduct inter-rater assessment of our gastroenterologist readers (although extensive work has been done with their radiology counterparts^6^^,^^10^^,^^12^^,^^13^^,^^14^) which could have introduced bias as could have been exposed to the magnitude of change and potential 'enthusiasm' for having a new measurement tool. We also acknowledge that the study did in part take place during the COVID pandemic, potentially influencing clinical practice and steer towards remote care such that patients attending hospitals may have had more severe disease. Finally, we acknowledge that no clinical changes were made directly as a result of GIQuant. Principally, we did not ask nor mandate a change in management as part of this trial due to the novelty of the biomarker and focus of this work on evaluation of impression. Treatment changes come from confidence, confidence comes from use and use from availability. As such, this study is a step on the path towards biomarker uptake and supports future interventional trials as part of a treat-to-target approach.

With the rise in popularity of intestinal ultrasound (IUS) how should we interpret these findings within the wider IBD monitoring landscape? To recap, IUS is inexpensive, widely available and can be performed during a patient consultation to provide actionable point of care information. In contrast, MRE is perceived as expensive, waiting times are long in most countries and, due to the need for bowel preparation, the test is unpleasant for patients. Perhaps most importantly, high quality studies including METRIC^26^ showed that the two modalities were similar in terms of sensitivity and specificity. Against this backdrop, we must ask ourselves if MRE is still relevant in 2026 and where do the findings of CONTEXT take us? First, we need to accept that MRE for Crohn’s Disease has not evolved in 20 years and now lags behind other medical specialties in terms of utility—it’s an extravagant way to collect a bowel wall thickness measurement. We continue to operate a 'one size fits all' approach with a potpourri of imaging sequences in a largely subjective manner. Why should a person with stricturing disease receive the same protocol as a new diagnosis or a simple follow up of a terminal ileal lesion? Shorter, more focused scans with less bowel preparation without intravenous contrast or spasmolytic should be a priority for a modern IBD service. Indeed we saw this in our study with all but one of the sites now having dropped standard IV contrast imaging shortening overall scan times. Second, we have not seen advances in quantitative imaging in Crohn’s that have taken off elsewhere in oncology, cardiology, neurology etc. to elevate the diagnostic contribution of this powerful modality. GIQuant is a clinically available step that shows how MRI, as a tool, will differentiate itself and ideally complement tests like IUS. We should also remember, cardiac MRI and echocardiography co-exist and it should always be about using the *right tool for the job*. For now, and together with published data, we can be confident that technically, mMRI assessed with GIQuant is robust methodologically in both adults and children and practicable alongside clinical practice supporting the advance of the approach. These data support the relevance of MRE as the monitoring landscape evolves.

In conclusion, GIQuant as a measure of motility MRI appears robust in clinical use as a method to assess small bowel Crohn’s activity with a demonstrable impact on the evaluation of disease status across time points by U.K. Gastroenterologists.

## Statement on conflicts of interest

AM—Employed by Motilent and holds shares in Motilent. Consultancy for J&J, Astra Zeneca, Dr Falk & Takeda. IN & LB—Employed by Motilent. JL—Speaker and consultancy fees: Abbvie, Alpha Sigma, BioHit, Bristol-Myers Squibb, Celltrion, Eli Lilly, Ferring, Johnson & Johnson, MSD, Pfizer and Takeda and research grants, Galapagos and Takeda. SAT—NIHR Senior Investigator, Holds Motilent shares and consultancy for Astra Zeneca and grant holder with Takeda. GB—Consultancy for Motilent & Alimentiv. KVP—KVP reports payment or honoraria for lectures, presentations, speakers bureaus, manuscript writing or educational events from AbbVie, DrFalk, Janssen, PreddictImmune and Takeda; support for attending meetings or travel from AbbVie, Ferring, Janssen and Tillotts; and participation on a data safety monitoring board or advisory board for AbbVie, Galapagos and Janssen. GM—Consultancy/speaker fees: Pfizer, Jansen, Takeda, Bristol Myers Squibb, Abbvie, Alimentiv Inc, Satisfai, Health, Dova Health. Research grants: Astra Zeneca, Pfizer, Jansen, Bristol Myers Squibb. JB, NP, AO, SB, NO, VJ, SV, CC, RM, TS, SU, JR, AK, AW, PG, AS, DT, HF, WW, PH, JY, MH report no relevant conflicts of interest.

## Statement on author contribution

1. The conception and design of the study (GM, SAT, AM, HF, LB). Acquisition of data (JB, NP, AO, SB, NO, VJ, SV, CC, RM, GB, TS, SU, JR, AK, AW, PG, AS, DT, HF, WW, PH, JY, MH, SAT & GM). Analysis and interpretation of data (SAT, GM, AM, IN, HF, JL, JB, GB)
2. drafting the article or revising it critically for important intellectual content (AM, JL, JN, GM, SAT)
3. final approval of the version to be submitted (GM, AM & SAT)

No writing help nor AI was used in the preparation of this manuscript.

## Funding statement

This project NIHR201000 (PI Alex Menys) is funded by the Invention for Innovation (i4i) programme, Product Development Awards an NIHR programme. The views expressed in this publication are those of the author(s) and not necessarily those of the NIHR or the Department of Health and Social Care.

## Data availability statement

Data, analytic methods, and study materials will be made available to other researchers on reasonable request.

## Figures and Tables

**Figure A1.1:**
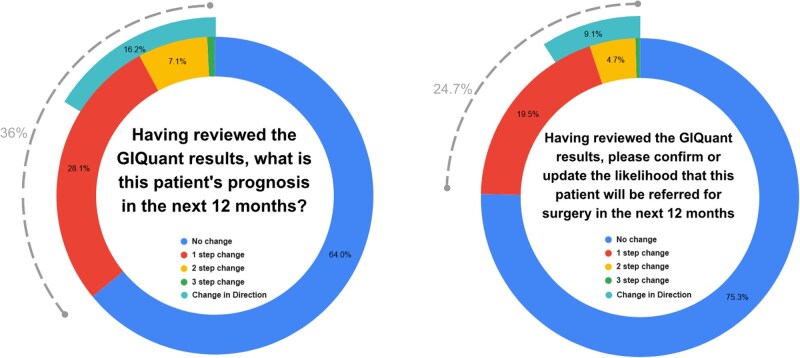
Change in clinicians' 12 months prognosis after reviewing GIQuant results. A1.2 change in clinicians' surgical referral likelihood after reviewing GIQuant results.

## Appendix 1

**Table A1.1.** Changes in impression around i) disease status, ii) 12 months prognosis and iii) likelihood of surgery within the next 12 months after reviewing the GIQuant results.

	Diagnosis	Prognosis	Surgery likelihood
**Total cases**	253	253	215*
**Change in opinion**	120 (47.4%)	91 (36.0%)	53 (24.7%)
**No change**	133	162	162
**Opinion reversal**	55 (21.7%)	41 (16.2%)	23 (10.7%)
**1 step change**	87	71	42
**2 step change**	24	18	10
**3 step change**	7	2	1
**4 step change**	2	0	0

***38 already been referred to surgery which were excluded**.

## Appendix 2

**Participant Information CRF** 

Please select your age range

- □ 25–34
- □ 35–44
- □ 45–54
- □ 55–65
- □ 65–70

Please select your role

- □ Radiologist
- □ Gastroenterologist

Please select your position (if Gastroenterologist)

- □ Consultant Gastroenterologist
- □ IBD Fellow

How many years have you held this position?

How many endoscopies do you perform each month?

What proportion (%) of those endoscopies are for small bowel Crohn’s disease?

**Experience using GIQuant** 

How many patients with GIQuant results have you personally reported or reviewed as part of your Research practice?

How many patients with GIQuant results have you personally reported or reviewed as part of your Clinical practice?

Conflict of interest

Do you have any relevant financial or non-financial interests and relationships that might be considered likely to affect the interpretation of their findings or that editors, reviewers, or readers might reasonably wish to know?

- □ Yes
- □ No

Please state the nature of any potential conflicts of interest

**Gastroenterology eCRF** 

**Eligibility:** 

Please select options below to confirm that the patient you are reviewing is eligible for this study.

If the patient is eligible additional tabs will appear for you to complete.

If the patient is not eligible then you should not enter any data and move onto the next patient on your clinic list.

**Patient age on the date of the second MR scan** 

- □ <16
- □ 16–25
- □ 26–35
- □ 36–45
- □ >45

**Patient sex** 

- □ Female
- □ Male
- □ Other

**Does this patient have documented small bowel Crohn’s disease?** 

- □ Yes
- □ No
- □ Not known

**Does this patient have documented small bowel Crohn’s disease?** 

- □ Yes
- □ No
- □ Not known

**Patient details** 

**Disease duration** 

- □ New diagnosis
- □ <12 months
- □ 1–2 years
- □ 3–4 years
- □ >4 years

**Smoking status** 

- □ Never Smoked
- □ Ex-Smoker
- □ Current Smoker
- □ Not known

**Current treatment (at the time of their 2nd MRE scan)** 

- □ Yes
- □ No

**- (If Yes), Please select all that apply*** 

- □ Corticosteroids
- □ Thiopurines
- □ Methotrexate
- □ Biologics
- □ Another advanced therapy (e.g. JAK inhibitor)

**Current biologics or advanced therapy (at the time of their 2nd MRE scan)** 

**- Drug** 

- □ Infliximab
- □ Adalimumab
- □ Ustekinumab
- □ Vedolizumab
- □ Upadacitinib
- □ Other

**Dose (mg) Frequency (interval in weeks) Duration (months)** 

**Clinical details** 

**If patient age on the eligibility from was > 16** 

**Age at diagnosis** (Montreal classification)

- □ <17 (A1)
- □ 17–40 (A2)
- □ >40 (A3)
- □ Not known

**Location of disease** (Montreal classification)

- □ Ileal (L1)
- □ Ileo-colonic (L3)
- □ Not known

**Upper digestive modifier L4** (Montreal classification)

- □ Yes
- □ No
- □ Not known

**Behaviour of disease** (Montreal classification)

- □ Non-stricturing, non-penetrating (B1)
- □ Stricturing (B2)
- □ Penetrating (B3)
- □ Not known

**If patient age on the eligibility from was < 16** 

**Age at diagnosis (Paris classification)** 

- □ 0 - <10 (A1a)
- □ 10 - <17 (A1b)

**Location of disease (Paris classification)** 

- □ Distal 1/3 ileum ± limited caecal (L1)
- □ Ileocolonic (L3)
- □ Not known

**Upper digestive modifier L4 (Paris classification)** 

- □ Upper disease proximal to the Ligament of Treitz (L4a)
- □ Upper disease distal to the Ligament of Treitz (L4b)

**Behaviour of disease (Paris classification)** 

- □ Non-stricturing, non-penetrating (B1)
- □ Stricturing (B2)
- □ Penetrating (B3)
- □ Penetrating and structuring (B2B3)

**Perianal disease modifier (Montreal & Paris classification)** 

- □ Yes
- □ No
- □ Not known

**Growth (Paris classification)** 

- □ No evidence of growth delay (G0)
- □ Growth delay (G1)

**Previous bowel surgery?** 

- □ No surgery
- □ Single surgery
- □ Multiple surgeries

**Date of most recent surgery (month)** 

**Date of most recent surgery (year)** 

**Type of surgery** 

**Conventional investigations (without GIQuant)** 

The following section should be completed based on conventional investigations including the standard MRE report at two time-points.

In order to estimate resource utilisation, we will ask you to record the tests that patients have had within 3 months of each MRE scan.

DO NOT view the GIQuant results at the end of the MRE report until you have completed this section.

**Please provide information about the tests available at each time-point** 

**Table:**

MR Enterography—year	First time-point	Second time-point
**MR Enterography—month**	**What year was their previous MRE performed?**	**What year was their current MRE performed?**
**MR Enterography—indication**	**What month was their previous scan performed?**	**What month was their current scan performed?**
**Standard tests**	**What was the indication for the previous MRE?** Crohn’s disease—new diagnosis Crohn’s disease—post-treatment follow-up Crohn’s disease—suspected relapse Other—related to Crohn’s disease	**What was the indication for the current MRE?*** Crohn’s disease—new diagnosis Crohn’s disease—post-treatment follow-up Crohn’s disease—suspected relapse Other—related to Crohn’s disease
**Other tests**	**Performed within 3 months of previous MRE?** Colonoscopy CT enterography Capsule endoscopy Harvey Bradshaw Index Blood tests (e.g., CRP) Faecal Calprotectin Abdominal Ultrasound Enteroscopy Other	**Performed within 3 months of current MRE?*** Colonoscopy CT enterography Capsule endoscopy Harvey Bradshaw Index Blood tests (e.g., CRP) Faecal Calprotectin Abdominal Ultrasound Enteroscopy Other
	**If other, please state:**	**If other, please state:**

**OVERALL ASSESSMENT—Standard tests** 

**Based on all available standard tests, how would you describe the patient’s change in status between the dates below?** 

- □ Much worse
- □ Somewhat worse
- □ Slightly worse
- □ Slightly better
- □ Somewhat better
- □ Much better

**Start date: ___/____/____End date: ___/____/____** 

**Based on standard tests, how do you intend to manage this patient?** 

**Patient is on no medication for active Crohn’s and none will be added** 

- □ Yes
- □ No

**Continue with current medication and dose for active Crohn’s** 

- □ Yes
- □ No

**Reduce dose of current medication for active Crohn’s** 

- □ Yes
- □ No

**Increase dose of current medication for active Crohn’s** 

- □ Yes
- □ No

**Stop current medication for active Crohn’s** 

- □ Yes
- □ No

**Initiation of corticosteroids** 

- □ Yes
- □ No

**Advanced therapy will now be started for active Crohn’s disease** 

- □ Yes
- □ No

**Switch within biological class of medication** 

- □ Yes
- □ No

**Change in biological class of medication** 

- □ Yes
- □ No

**Immunotherapy will be started** 

- □ Yes
- □ No

**Refer for surgical therapy** 

- □ Yes
- □ No

**Other?** 

- □ Yes
- □ No

**If other, please state** 

**THERAPEUTIC CONFIDENCE—Standard tests** 

**Based on this management plan, what is this patient’s prognosis in the next 12 months?** 

- □ Much worse
- □ Somewhat worse
- □ Slightly worse
- □ Slightly better
- □ Somewhat better
- □ Much better

**Based on this management plan, what is the likelihood of this patient being referred for surgery in the next 12 months?** 

- □ Definitely not
- □ Probably not
- □ Possibly not
- □ Possibly
- □ Probably
- □ Definitely

**Conventional investigations (with GIQuant)** 

The following section should be completed based on conventional investigations, including the standard MR Enterography (MRE) report and GIQuant results, at two time-points.

The GIQuant score should be at the very end of the MRE report or may have been reported separately as an addendum.

If a patient has an MRE report at either time-point that is missing a GIQuant score, you should contact a local radiologist to request an addendum with the GIQuant score for any equivocal or abnormal disease segments.

**Please provide details of all reported GIQuant scores at each time-point** 

**Table:**

Site of small bowel disease	Has GIQuant score been reported at both time-points?	GIQuant score for previous MRE	GIQuant score for current MRE	Change over time
**Duodenum** **Jejunum** **Ileum** **Terminal ileum** **Other location** **Not known** **No GIQuant score reported**	No—for PREVIOUS scan only No—for CURRENT scan only Yes—for BOTH time-points			*Calculated field*

**OVERALL ASSESSMENT—GIQuant** 

**Based on standard tests, you described the change in this patient’s status between the two MRE examinations as:** 

*display previously stated answer*

**Having reviewed the GIQuant results, how would you describe the patient’s change in status between the dates below?** 

- □ Much worse
- □ Somewhat worse
- □ Slightly worse
- □ Slightly better
- □ Somewhat better
- □ Much better

**THERAPEUTIC CONFIDENCE—GIQuant** 

**Based on standard tests, you thought the patient’s status would get:** 

*display previously stated answer*

**Having reviewed the GIQuant results, what is this patient’s prognosis in the next 12 months** 

- □ Much worse
- □ Somewhat worse
- □ Slightly worse
- □ Slightly better
- □ Somewhat better
- □ Much better

**Based on standard tests, you thought the likelihood of this patient being referred for surgery in the next 12 months was:** 

*display previously stated answer*

**Having reviewed the GIQuant results, please confirm or update the likelihood that this patient will be referred for surgery in the next 12 months** 

- □ Definitely not
- □ Probably not
- □ Possibly not
- □ Possibly
- □ Probably
- □ Definitely

**GIQuant results** 

**Having reviewed the GIQuant results, would you theoretically change your management plan?** 

**Patient is on no medication for active Crohn’s and none will be added** 

*display previously stated answer*

- □ Yes
- □ No

**Continue with current medication and dose for active Crohn’s** 

*display previously stated answer*

- □ Yes
- □ No

**Reduce dose of current medication for active Crohn’s** 

*display previously stated answer*

- □ Yes
- □ No

**Increase dose of current medication for active Crohn’s** 

*display previously stated answer*

- □ Yes
- □ No

**Stop current medication for active Crohn’s** 

*display previously stated answer*

- □ Yes
- □ No

**Initiation of corticosteroids** 

*display previously stated answer*

- □ Yes
- □ No

**Advanced therapy will now be started for active Crohn’s disease** 

*display previously stated answer*

- □ Yes
- □ No

**Switch within biological class of medication** 

*display previously stated answer*

- □ Yes
- □ No

**Change in biological class of medication** 

*display previously stated answer*

- □ Yes
- □ No

**Immunotherapy will be started** 

*display previously stated answer*

- □ Yes
- □ No

**Refer for surgical therapy** 

*display previously stated answer*

- □ Yes
- □ No

**Other?** 

*display previously stated answer*

- □ Yes
- □ No

**If other, please state** 

*display previously stated answer*

## Appendix 3

**Table:**

Parameter	Site 1	Site 2				Site 3	Site 4	Site 5		Site 6
**Acquisition plane**	Coronal	Coronal				Coronal	Coronal	Coronal		Coronal
**Temporal resolution (images per second)**	1	1	0.9	1	1.2	1	1	1	0.8	1
**Number of time points**	20	20	18	20	18	15	20	20	100	20
**Number of slice positions**	Variable to cover abdomen	Variable to cover abdomen	Variable to cover abdomen	Grouped, multi-slice series (10 slices)	Grouped, multi-slice series (10 slices)	Variable to cover abdomen	Variable to cover abdomen	15	6 (placed over TI)	Variable to cover abdomen
**Sequence name**	BFFE	BFFE	TruFISP	TruFISP	BFFE	FIESTA	TruFISP	BFFE	TruFISP	TruFISP
**Slice thickness** **(cm)**	1	1	1	1	1	1	1	1	1	1
**Brathhold (BH)/free breathing (FB)**	BH	BH	BH	BH	BH	BH	BH	BH	FB	BH
**Manufacturer**	Philips	Philips	Siemens	Siemens	Phillips	GE	Siemens	Phillips	Siemens	Siemens
**Field strength** **(T)**	3	3	1.5	1.5	1.5	1.5	1.5	3	1.5	1.5
**Fast before prep?**	Yes 3 hours	Yes 3 hours	Yes 3 hours	Yes 3 hours	Yes 3 hours	Yes 3 hours	Yes over 3 hours	Yes over 3 hours	Yes over 3 hours	Yes 3 hours
**Preparation**	2.5% Mannitol	2.5% Mannitol	2.5% Mannitol	2.5% Mannitol	2.5% Mannitol	2%Mannitol	2% Mannitol	VoLumen	VoLumen	2.5% Mannitol
**Volume & timing of prep**	1000 Ml 1 h before scan	1000ML to 2000ML depending on habitus 1 h before scan	1000ML to 2000ML depending on habitus 1 h before scan	1000ML to 2000ML depending on habitus 1 h before scan	1000ML to 2000ML depending on habitus 1 h before scan	1000ML to 2000ML depending on habitus 1 h before scan	1000ML to 2000ML depending on habitus 2 h before scan	1 to 2 L depending on habitus	1 to 2 L depending on habitus	1000ML to 2000ML depending on habitus 1 h before scan
